# Synthesis of New Naphtho[2,3-*f*]quinoxaline-2,7,12(1*H*)-trione and Anthra-9,10-quinone Dyes from Furan-2,3-diones

**DOI:** 10.3390/molecules14041429

**Published:** 2009-04-02

**Authors:** Şevket Hakan Üngören

**Keywords:** Naphtho[2,3-*f*]quinoxaline-2,7,12(1*H*)-triones, Anthra-9,10-quinones, Furan-2,3-diones, Diaminoanthra-9,10-quinone, Dye modification, Vat dyes.

## Abstract

Novel naphtho[2,3-*f*]quinoxaline-2,7,12(1*H*)-trione and anthra-9,10-quinone dyes were synthesized in good yield from furan-2,3-diones using 1,2-diaminoanthra-9,10-quinone and 1,4-diaminoanthra-9,10-quinone. The chromophores were characterized by molecular spectroscopy methods.

## 1. Introduction

**Figure 1:**
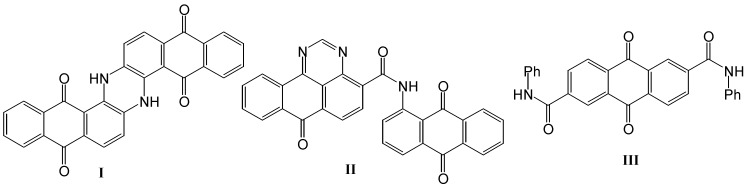
Some commercial vat dyes.

Anthra-9,10-quinones and their condensed derivatives with heterocycles such as indanthrone (Pigment Blue 60, **I**), anthrapyrimidine (Pigment Yellow 108, **II**) and Vat Yellow 3 (**III**) (Figure 1) possess brilliant hues and very good fastness and represent an important group of vat dyes for the textile industry [1,2]. In addition to these properties, some anthra-9,10-quinone dyes are widely used in other fields, such as in medicine and food chemistry [3] and high-technology systems [4]. Consequently, anthra-9,10-quinones are interesting compounds from the viewpoint of both their reactions and applications.

Furan-2,3-diones (**1**) have been shown to be very useful synthons for the preparation of various heterocycles. These compounds show typical carbonyl and lactone reactions, depending on the structures of the nucleophiles involved [5,6,7,8]. For example, furan-2,3-diones undergo cyclocondensations with 1,2-diamines to provide the corresponding quinoxalines and aromatic amines react with furan-2,3-diones to give the corresponding Schiff bases and pyrrole-2,3-dione derivatives, depending on the reaction times and temperature [5,9].

Furan-2,3-diones (**1**) can offer many possibilities for the construction of various heterocyclic dyes including the new naphtho[2,3-*f*]quinoxaline and anthra-9,10-quinone derivatives reported herein. In this work we present new anthra-9,10-quinone (**2**) and naphtho[2,3-*f*]quinoxaline (**3**) dyes derived from the reactions of some furan-2,3-diones with 1,2-diaminoanthra-9,10-quinone (**1,2-DAAQ**) and 1,4-diaminoanthra-9,10-quinone (**1,4-DAAQ**), acting as bifunctional nucleophiles. 

## 2. Results and Discussion

**Scheme 1:**
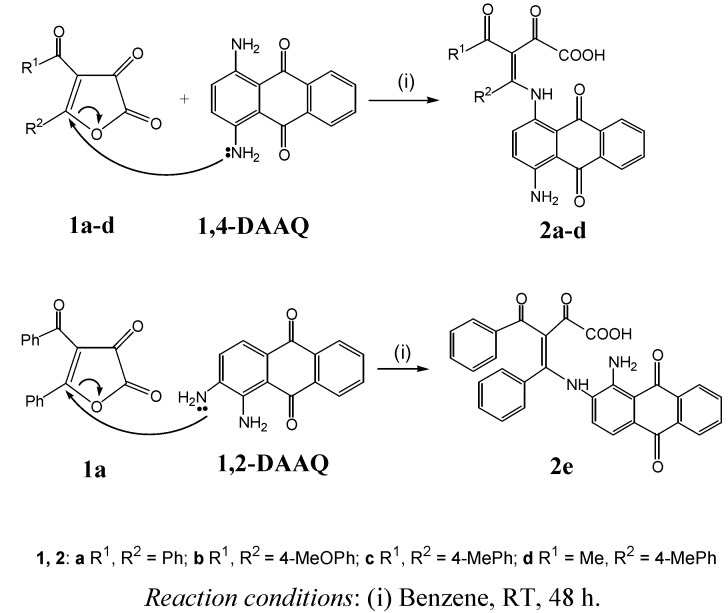
Synthesis of compounds **2**.

Furan-2,3-dione starting materials **1a-f** were prepared according to the literature [10,11,12,13]. The C_5_ atom of compounds **1a-d** smoothly reacted with the amino group of **1,2-DAAQ** and **1,4-DAAQ** to give compoubds **2** under mild conditions and in high yields (75-90%, Scheme 1). 

*Reaction conditions*: (i) Benzene, RT, 48 h.

**Scheme 2:**
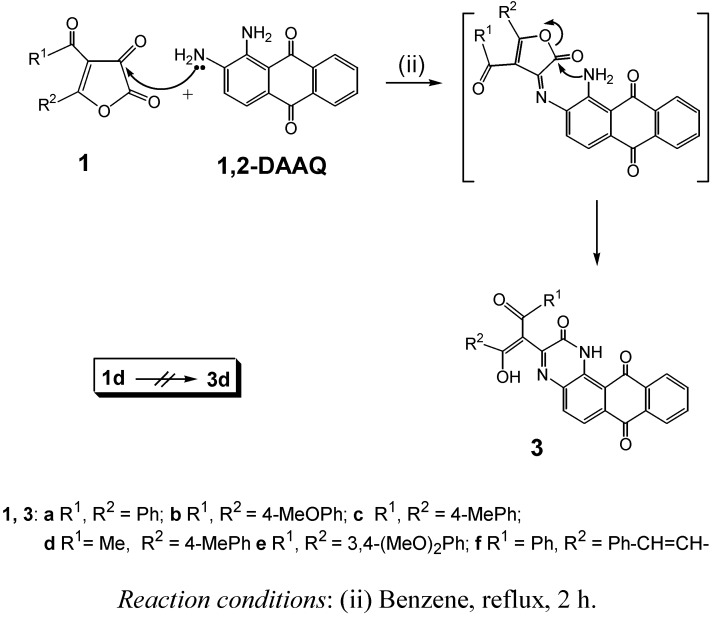
Synthesis of compounds **3**.

Due to the greater reactivity of the amino group attached to the C_2_ atom of **1,2-DAAQ**, compared with the amino group attached to the C_1_ atom of **1,2-DAAQ**, **1,2-DAAQ** was modified from the amino group attached to C_2_-position of **1,2-DAAQ** to give **2e**. On the other hand, the amino group attached to the C_2_ atom of **1,2-DAAQ** did not react with the C_5_ atom of **1a** at higher temperature, but reacted with the C_3_ atom of **1a** by forming a Schiff base, which was not isolated (as outlined in Scheme 2). Through attack of the second amino group on the lactone carbonyl group, ring opening occurs. The reactions of **1,2-DAAQ** with **1b,c,e,f** run via the samereaction pathways to give **3** in nearly quantitative yields of 90-96% in boiling benzene. This proposed mechanism is similar to that reported in the literature for the reaction pathways of furan-2,3-diones with 1,2-diamino nucleophiles [9,14]. 

*Reaction conditions*: (ii) Benzene, reflux, 2 h.

**Scheme 3:**
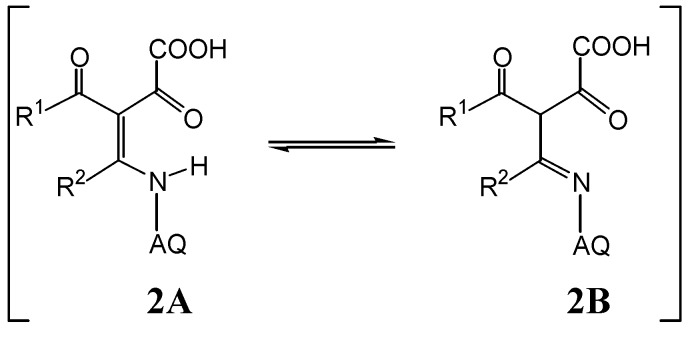
Tautomeric forms (**2A** and **2B**) of **2a-e** in DMSO-*d_6_*.

The structures of **2** and **3** were confirmed by spectroscopic data and agree with those found for similar compounds [4,9,14,15]. In the NMR spectra, the methine proton signal (low intensity) also revealed that compound **2** occurs as tautomers (**2A** and **2B**), with tautomer **2B** as a minor contributor in DMSO-*d_6_* solutions (Scheme 3). The ^13^C-NMR spectroscopic data of **2** also agree with the proposed tautomeric structures.

**Scheme 4:**
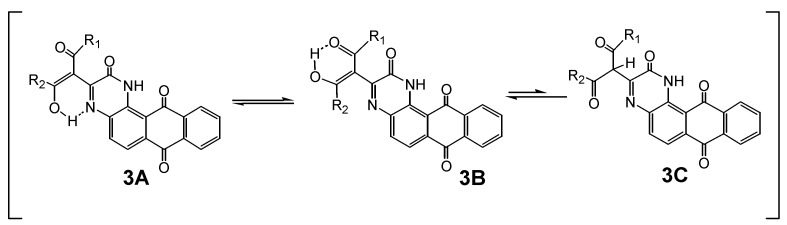
Tautomeric forms (**3A-C**) of **3a,f** in DMSO-*d_6_*.

There was indication of tautomeric forms **3A** (and **3B** as a minor contributor) in **3a,f** (but not **3b,c,e)** in their ^1^H-NMR spectra in DMSO-*d_6_* solution (Scheme 4). However, there was no signal for the methine proton belonging to the tautomer **3C** in DMSO-*d_6_* solution. The ^13^C-NMR spectra of **3** could not be recorded due to its very low solubility in organic solvents, but the condensation was verified by the detection of the [MH^+^] and [MH^+^-H_2_O] signals.

**Figure 2:**
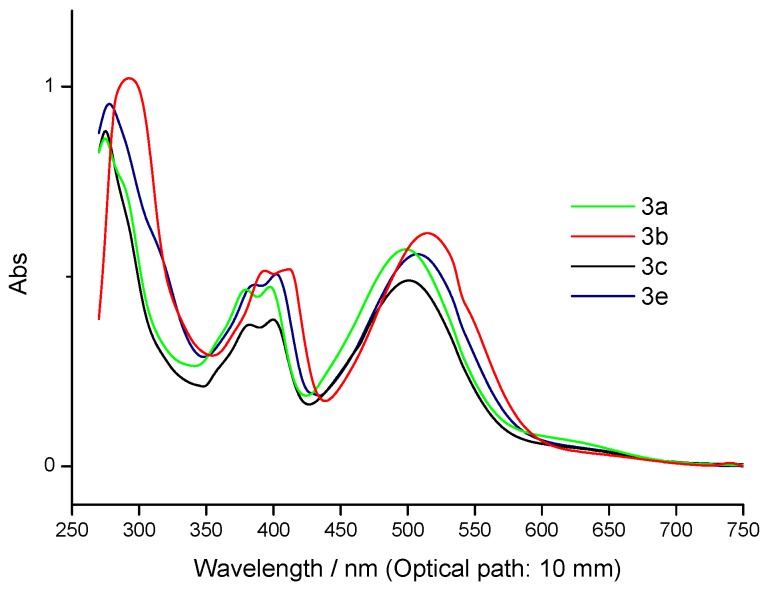
UV-Vis spectra of compound **3** in DMF.

The UV-vis absorption spectra of **3a-c,e** in DMF at a concentration of 3.333 x 10^-5 ^mol/L are shown in Figure 2. The results are also listed in Table 1. In the visible region, the molar absorption coefficients and the absorption maxima of compounds **3** were observed in the range 1.119x10^4 ^to 1.545x10^4 ^dm^3^ mol^-1 ^cm^-1^ (380-394 nm), 1.161x10^4 ^to 1.557x10^4 ^(397-412 nm), 1.467x10^4 ^to 1.842x10^4 ^(498-515 nm), there being no significant difference between them. The visible absorption spectrum is dominated by the characteristic anthraquinone bands in the region 380-400 nm [15].

**Table 1:** UV-Vis spectral data of **3**.

Compounds	UV-Vis			
	λ_1-4max_(*nm*),			
	ε_1-4_ (*liter mol^-1^ cm^-1^*)			
**3a**	274,	380,	397,	498,
	2.592x10^4^	1.398x10^4^	1.416x10^4^	1.716x10^4^
**3b**	293,	394,	412,	515,
	3.066x10^4^	1.545x10^4^	1.557x10^4^	1.842x10^4^
**3c**	275,	382,	400,	501,
	2.652x10^4^	1.119x10^4^	1.161x10^4^	1.467x10^4^
**3e**	278,	386,	402,	507,
	2.865x10^4^	1.434x10^4^	1.518x10^4^	1.677x10^4^

## 3. Experimental Section

### 3.1. General

Solvents were purchased from Merck and Carlo Erba. Diaminoanthra-9,10-quinones were purchased from Aldrich and used without further purification. ^1^H- and ^13^C-NMR spectra were recorded using a Bruker Ultrashield spectrometer operating at 300.13 MHz (^1^H) and 75.47 MHz (^13^C). UV-vis spectra: Shimadzu UV-1280 spectrophotometer. IR Spectra: Jasco Plus Model 460 FTIR spectrometer; in cm^-1^. Elemental analyses (C, H, N) were performed using a Leco CHNS-O 932. Melting points were measured with an Electrothermal 9200 apparatus. GC/MS measurements were performed using a gas chromatograph (Model 6890 Series)-mass selective detector (Model 5973N) system (Agilent Technologies).

### 3.2. General Procedure for the Preparation of Compounds ***2a**-**e***

Compound **1** (1 mmol) and **1,2-/1,4-DAAQ** (1 mmol) in benzene (60 mL) was stirred for 48 h. The precipitate **2** was filtered off and recrystallized from DMF.

*4-[(4-Amino-9,10-dioxo-9,10-dihydroanthracen-1-yl)amino]-3-benzoyl-2-oxo-4-phenylbut-3-enoic acid* (**2a**): Obtained from **1a** (0.278 g, 1 mmol) and **1,4-DAAQ** (0.238 g, 1 mmol). Mp. 195-196 °C as dark-blue crystals (0.415 g, 80%); ^1^H-NMR (DMSO-*d_6_*): δ = 13.50 (1H, s, NH), 8.77-6.35 (16H, m, aromatic), 7.16 (2H, s, NH_2_, br), 4.89 (Ar-CO-CH-CO, tautomer **B**), COOH not observed; ^13^C-NMR (DMSO-*d_6_*): δ = 193.29, 192.53, 188.81, 184.79, 183.47, 181.49 (C=O), 157.60, 152.96, 150.81, 147.19, 139.82, 137.98, 135.72, 135.04, 134.82, 134.55, 134.12, 134.05, 133.95, 133.84, 133.71, 133.47, 133.08, 132.68, 132.20, 132.07, 130.92, 130.47, 129.98, 129.70, 129.57, 129.40, 129.29, 129.12, 129.04, 128.86, 128.69, 128.15, 128.00, 127.87, 127.06, 126.46, 126.20, 124.43, 120.96, 118.32, 113.17, 111.94, 110.63, 110.20, 107.97, 93.69 (C=C, arom., aliph.), 64.05 (-CH-); IR (KBr) ν 3450 (OH, br); 3445, 3427 (NH_2_); 3302 (NH); 1724, 1690, 1670, 1636 cm^-1^ (C=O); Anal. Calcd for C_31_H_20_N_2_O_6_ (516.5): C, 72.09; H, 3.90; N, 5.42. Found: C, 72.26; H, 3.88; N, 5.28. 

*4-[(4-Amino-9,10-dioxo-9,10-dihydroanthracen-1-yl)amino]-3-(4-methoxybenzoyl)-4-(4-methoxy-phenyl)-2-oxobut-3-enoic acid* (**2b**): Obtained from **1b** (0.338 g, 1 mmol) and **1,4-DAAQ** (0.238 g, 1 mmol). Mp 215-216 °C as black crystals (0.503 g, 87%); ^1^H-NMR (DMSO-*d_6_*): δ = 13.55, 13.48 (1H, d, NH), 8.97-6.70 (14H, m, aromatic), 7.07 (2H, s, NH_2_, br), 4.72 (Ar-CO-CH-CO, tautomer **B**), 3.86, 3.79 (6H, s, OCH_3_), COO*H* not observed;^ 13^C-NMR (DMSO-*d_6_*): δ = 192.73, 191.76, 191.50, 184.62, 183.51, 181.49 (C=O, C=N), 164.44, 163.45, 162.77, 161.41 (C=C-OCH_3_, tautomer **A** and **B** ), 157.78, 147.20, 135.07, 134.56, 134.12, 133.98, 133.08, 132.70, 131.79, 131.72, 131.36, 131.04, 129.91, 129.29, 128.72, 127.59, 127.08, 126.47, 126.21, 114.77, 114.56, 113.54, 107.97, 91.94 (C=C, arom., aliph.), 65.24 (-CH-) 36.24, 34.64, 31.23 (OCH_3_); IR (KBr) ν 3455 (OH, br); 3425 (NH_2_); 3300 (NH); 1726, 1686, 1664, 1638 cm^-1^ (C=O); Anal. Calcd for C_33_H_24_N_2_O_8_ (576.6): C, 68.75; H, 4.20; N, 4.86. Found: C, 68.84; H, 4.40; N, 4.81. 

*4-[(4-Amino-9,10-dioxo-9,10-dihydroanthracen-1-yl)amino]-3-(4-methylbenzoyl)-4-(4-methylphenyl)-2-oxobut-3-enoic acid* (**2c**): Obtained from **1c** (0.306 g, 1 mmol) and **1,4-DAAQ** (0.238 g, 1 mmol). Mp 220-221°C as navy-blue crystals (0.493 g, 90%); ^1^H-NMR (DMSO-*d_6_*): δ = 13.54, 13.49 (1H, d, NH), 8.77-6.62 (14H, m, aromatic), 7.58 (2H, s, NH_2_, br), 4.79 (Ar-CO-CH-CO, tautomer **B**), 3.45 (1H, s, COO*H*, br), 2.41, 2.31 (14H, s, CH_3_); ^13^C-NMR (DMSO-*d_6_*): δ = 192.95, 192.69, 192.61, 187.08, 183.55, 181.49 (C=O, C=N), 157.69, 150.24, 147.21, 145.48, 135.11, 134.56, 134.00, 133.90, 133.33, 133.15, 132.70, 130.99, 130.09, 129.87, 129.79, 129.46, 129.38, 129.30, 128.80, 128.17, 127.88, 127.18, 127.11, 126.49, 126.21, 118.36, 110.22, 107.95 (C=C, arom., alph.), 63.83 (-CH-), 21.71, 21.55 (CH_3_); IR (KBr) ν 3480 (OH, br); 3442 (NH_2_); 3308 (NH); 1725, 1689, 1671, 1663, 1638 cm^-1^ (C=O); Anal. Calcd for C_33_H_24_N_2_O_6_ (544.6): C, 72.78; H, 4.44; N, 5.14. Found: C, 72.92; H, 4.35; N, 4.96.

*3-Acetyl-4-[(4-amino-9,10-dioxo-9,10-dihydroanthracen-1-yl)amino]-4-(4-methylphenyl)-2-oxobut-3-enoic acid* (**2d**): Obtained from **1d** (0.230 g, 1 mmol) and **1,4-DAAQ** (0.238 g, 1 mmol). Mp 174-175 °C as brown crystals (0.419 g, 90%); ^1^H-NMR (DMSO-*d_6_*): δ = 13.54, 13.44 (1H, d, NH), 8.92-6.86 (10H, m, aromatic), 7.58 (2H, s, NH_2_, br), 4.64 (Ar-CO-CH-CO, tautomer **B**), 3.51 (1H, s, COO*H*, br), 2.39 (3H, s, COCH_3_), 2.22 (3H, s, C=C-CH_3_); ^13^C-NMR (DMSO-*d_6_*): δ = 200.05, 192.45, 186.70, 183.39, 181.47 (C=O, C=N), 152.97, 150.05, 147.19, 146.02, 136.21, 135.72, 134.92, 134.54, 133.92, 133.76, 133.09, 132.69, 131.21, 130.20, 130.04, 129.88, 129.70, 129.37, 129.27, 128.07, 127.00, 126.41, 126.20, 117.89, 116.76, 109.96, 107.95, 105.96 (C=C, arom., alph.), 36.25 (COCH_3_), 21.59 (CH_3_); IR (KBr) ν 3475 (OH, br); 3432 (NH_2_); 3300 (NH); 1734, 1685, 1666, 1637, 1638 cm^-1^ (C=O); Anal. Calcd for C_27_H_20_N_2_O_6_ (468.4): C, 69.22; H, 4.30; N, 5.98. Found: C, 69.16; H, 4.15; N, 5.75. 

*4-[(1-Amino-9,10-dioxo-9,10-dihydroanthracen-2-yl)amino]-3-benzoyl-2-oxo-4-phenylbut-3-enoic acid* (**2e**): Obtained from **1a** (0.278 g, 1 mmol) and **1,2-DAAQ** (0.238 g, 1 mmol). Mp 255-256 °C as brown crystals (0.386 g, 75%); ^1^H-NMR (DMSO-*d_6_*): δ = 15.23 (1H, s, NH), 8.31-6.94 (16H, m, aromatic), 7.16 (2H, s, NH_2_, br), 3.38 (1H, s, br, COOH); ^13^C-NMR (DMSO-*d_6_*): δ = 194.27, 191.87, 188.77, 186.80, 185.32, 181.44 (C=O, C=N), 144.54, 142.51, 142.14, 134,56, 134.29, 133.54, 131,07, 129.63, 129.15, 128.43, 128.10, 127.86, 127.66, 127.51, 127.02, 126.60, 126.41, 125.63, 124.56, 124.18, 116.88, 109.01 (C=C, arom., alph.); IR (KBr) ν 3435 (OH, br, NH_2_); 3211 (NH); 1717, 1658, 1649, 1639 cm^-1^ (C=O); Anal. Calcd. for C_31_H_20_N_2_O_6_ (516.5): C, 72.09; H, 3.90; N, 5.42%. Found: C, 72.39; H, 3.72; N, 5.61.

### 3.3. General Procedure for the Preparation of Compounds ***3a-c,e,f***

Compound **1** (1 mmol) and **1,2-DAAQ** (1 mmol) in benzene (40 ml) was refluxed for 2 h. The red-coloured precipitate (**3**) was filtered off and recrystallized from DMSO. 

*3-(1-Benzoyl-2-oxo-2-phenylethyl)naphtho[2,3-f]quinoxaline-2,7,12(1H)-trione* (**3a**): Obtained from **1a** (0.278 g, 1 mmol) and **1,2-DAAQ** (0.238 g, 1 mmol). Mp 341-343 ^o^C; 0.517 g, 94%; ^1^H-NMR (DMSO-*d_6_*): δ = 15.70, 15.07 (1H, s, OH, br), 13.25, 12.27 (1H, s, NH-C=O, br), 8.33-6.79 (16H, m, Ar-H); IR (KBr) ν 3435 (OH), 3210 (NH), 3072, 2925 (C-H), 1694, 1661, 1651, 1621 cm^-1^ (C=O); MS (ESI)^+^: *m*/*z* 498 (1%) [M+H]^+^; Anal. Calcd for C_31_H_18_N_2_O_5_ (498.5): C, 74.69; H, 3.64; N, 5.62. Found: C, 74.78; H, 3.68; N, 5.60. 

*3-[1-(4-Methoxybenzoyl)-2-(4-methoxyphenyl)-2-oxoethyl]naphtho[2,3-f]quinoxaline-2,7,12(1H)-trione* (**3b**): Obtained from **1b** (0.338 g, 1 mmol) and 1,**2-DAAQ** (0.238 g, 1 mmol); Mp 347-349 ^o^C; 0.517 g, 92%; ^1^H-NMR (DMSO-*d_6_*): δ = 15.45 (1H, s, OH, br), 12.45 (1H, s, NHCO, br), 8.48-6.70 (14H, m, Ar-H), 3.80, 3.74 (6H, s, OCH_3_); IR (KBr) ν 3437 (OH), 3214 (NH), 3069, 2926 (C-H), 1695, 1664, 1654, 1638 cm^-1^ (C=O); MS (ESI)^+^: *m*/*z* 559 (5%) [M+H]^+^, *m*/*z* 541 (6%) [MH-H_2_O]^+^; Anal. Calcd for C_33_H_22_N_2_O_7_ (558.5): C, 70.96; H, 3.97; N, 5.02. Found: C, 70.79; H, 3.93; N, 5.17. 

*3-[1-(4-Methylbenzoyl)-2-(4-methylphenyl)-2-oxoethyl]naphtho[2,3-f]quinoxaline-2,7,12(1H)-trione* (**3c**): Obtained from **1c** (0.306 g, 1 mmol) and **1,2-DAAQ** (0.238 g, 1 mmol); Mp 315-317 ^o^C; 0.473 g, 90%; ^1^H-NMR (DMSO-*d_6_*): δ = 15.50 (1H, s, OH, br), 12.43 (1H, s, NHCO), 8.29-7.06 (14H, m, Ar-H), 2.34, 2.26 (6H, s, CH_3_); IR (KBr) *v* 3432 (OH), 3200 (NH), 3071, 2914 (C-H), 1693, 1663, 1656, 1638 cm^-1^ (C=O); MS (ESI)^+^: *m*/*z* 526 (13%) [M+H]^+^, *m*/*z* 525 (25%) [M]^+^; Anal. Calcd for C_33_H_22_N_2_O_5_ (526.5): C, 75.28; H, 4.21; N, 5.32. Found: C, 75.04; H, 4.29; N, 5.49. 

*3-[1-(3,4-Dimethoxybenzoyl)-2-(3,4-dimethoxyphenyl)-2-oxoethyl]naphtho[2,3-f]quinoxaline-2,7,12(1H)-trione* (**3e**): Obtained from **1e** (0.398 g, 1 mmol) and **1,2-DAAQ** (0.238 g, 1 mmol); Mp 309-311 ^o^C; 0.587 g, 95%; ^1^H-NMR (DMSO-*d_6_*): δ = 15.44 (1H, s, OH, br), 12.35 (1H, s, NHCO), 8.33-6.88 (12H, m, Ar-H), 3.81, 3.79, 3.75, 3.48 (12H, s, OCH_3_); IR (KBr) *v* 3436 (OH), 3253 (NH), 3075, 2932 (C-H), 1698, 1660, 1654, 1640 cm^-1^ (C=O); MS (ESI)^+^: *m*/*z* 619 (6%) [M+H]^+^, *m*/*z* 601 (2%) [MH-H_2_O]^+^; Anal. Calcd for C_35_H_26_N_2_O_9_ (618.6): C, 67.96; H, 4.24; N, 4.53. Found C, 68.17; H, 4.24; N, 4.60. 

*3-[(3E)-1-Benzoyl-2-oxo-4-phenylbut-3-en-1-yl]naphtho[2,3-f]quinoxaline-2,7,12(1H)-trione* (**3f**): Obtained from **1f** (0.304 g, 1 mmol) and **1,2-DAAQ** (0.238 g, 1 mmol); Mp 307-308 ^o^C; 0.472 g, 90%; ^1^H-NMR (DMSO-*d_6_*): δ = 15.64, 14.55 (1H, s, OH, br), 12.35, 12.11 (1H, s, NH), 8.28-6.82 (16H, m, Ar-H), 7.74 (1H, d, J = 15.4 Hz, CH=), 6.64 (1H, d, J = 15.3 Hz, CH=); IR (KBr) *v* 3435 (OH), 3213 (NH), 3062, 2853 (C-H), 1696, 1664, 1657, 1638 cm^-1^ (C=O); MS (ESI)^+^: *m*/*z* 525 (6%) [M+H]^+^, *m*/*z* 508 (3%) [MH-H_2_O]^+^; Anal. Calcd for C_33_H_20_N_2_O_5_ (524.5): C, 75.56; H, 3.84; N, 5.34. Found: C, 75.53; H, 3.80; N, 5.41.

## 4. Conclusions

We have designed and easily synthesized novel naphtho[2,3-*f*]quinoxaline-2,7,12(1*H*)-triones and anthra-9,10-quinones in good to excellent yields as potential vat dyes from furan-2,3-diones. Their spectroscopic properties in solution and in the solid state are reported. For commercial dye production, reactions with high yields and relatively straightforward chemistry are preferred. It was seen that furan-2,3-diones have good reactivity to meet these expectations. We believe that preparation of various heterocyclic dyes based on furan-2,3-dione chemistry might make a contribution to the development of high performance pigments. Performances of new dyes will be tested in future studies.
